# Ligand pathways in neuroglobin revealed by low-temperature photodissociation and docking experiments

**DOI:** 10.1107/S2052252519008157

**Published:** 2019-07-10

**Authors:** Chiara Ardiccioni, Alessandro Arcovito, Stefano Della Longa, Peter van der Linden, Dominique Bourgeois, Martin Weik, Linda Celeste Montemiglio, Carmelinda Savino, Giovanna Avella, Cécile Exertier, Philippe Carpentier, Thierry Prangé, Maurizio Brunori, Nathalie Colloc’h, Beatrice Vallone

**Affiliations:** a Department of Life and Environmental Sciences, New York–Marche Structural Biology Center (NY-MaSBiC), Polytechnic University of Marche, Ancona, Italy; b Istituto di Biochimica e Biochimica Clinica, Universitá Cattolica del Sacro Cuore, Largo Francesco Vito 1, 00168 Rome, Italy; c Fondazione Policlinico Universitario Agostino Gemelli–IRCCS, Largo Francesco Vito 1, 00168 Rome, Italy; d Department of Life, Health and Environmental Sciences, University of L’Aquila, 67100 L’Aquila, Italy; e European Synchrotron Radiation Facility (ESRF), 38043 Grenoble, France; f Partnership for Soft Condensed Matter (PSCM), 38043 Grenoble, France; g Université Grenoble Alpes, CEA, CNRS, IBS, 38000 Grenoble, France; h Department of Biochemical Sciences ‘A. Rossi Fanelli’, University of Rome Sapienza, Piazzale Aldo Moro 5, 00185 Rome, Italy; i Institute of Molecular Biology and Pathology, National Research Council, Piazzale Aldo Moro 5, 00185 Rome, Italy; j Istituto Pasteur–Fondazione Cenci Bolognetti, Department of Biochemical Sciences ‘A. Rossi Fanelli’, University of Rome Sapienza, Piazzale Aldo Moro 5, 00185 Rome, Italy; k Chemistry Department, Merck Serono S.p.A., Via Casilina 125, 00176 Rome, Italy; l CEA/DRF/BIG/CBM/BioCat LCBM CNRS UMR 5249, Université Grenoble Alpes, 38000 Grenoble, France; m CiTeCoM UMR 8038 CNRS, Université Paris Descartes, Paris, France; n ISTCT UMR 6030 CNRS Université de Caen Normandie CEA, CERVOxy Team, Centre Cyceron, Caen, France

**Keywords:** ultralow-temperature X-ray crystallography, XANES, heme protein, neuroglobin, oxygen binding, cryo-trapping, crystal microspectroscopy, neuroprotection, structural biology, CO photolysis, soak-and-freeze pressurization, structure determination, protein structure

## Abstract

A combined biophysical approach was applied to map gas-docking sites within murine neuroglobin (Ngb), revealing snapshots of events that might govern activity and dynamics in this unique hexacoordinate globin, which is most likely to be involved in gas-sensing in the central nervous system and for which a precise mechanism of action remains to be elucidated. The application of UV–visible microspectroscopy *in crystallo*, solution X-ray absorption near-edge spectroscopy and X-ray diffraction experiments at 15–40 K provided the structural characterization of an Ngb photolytic intermediate by cryo-trapping and allowed direct observation of the relocation of carbon monoxide within the distal heme pocket after photodissociation. Moreover, X-ray diffraction at 100 K under a high pressure of dioxygen, a physiological ligand of Ngb, unravelled the existence of a storage site for O_2_ in Ngb which coincides with Xe-III, a previously described docking site for xenon or krypton. Notably, no other secondary sites were observed under our experimental conditions.

## Abstract 2

Ultralow-temperature X-ray crystallography, *in crystallo* microspectroscopy and X-ray absorption spectroscopy were used to study the carbon monoxide photodissociation intermediate in neuroglobin. Moreover, X-ray crystallography under high O_2_ pressure allowed the identification of a novel storage site for dioxygen in hexacoordinate ferric neuroglobin.

## 1. Introduction   

Neuroglobin (Ngb) is a member of the globin protein family that is expressed in the brain (∼1 µ*M*) and the retina (∼100 µ*M*) of vertebrates (Burmester *et al.*, 2000 ▸). It is involved in the protection of the nervous tissue from ischemic damage *via* a still-elusive biochemical mechanism. Ngb is a highly conserved protein, with a mutation rate about threefold slower than those of myoglobin (Mb) and hemoglobin (Hb) (Burmester & Hankeln, 2004 ▸), suggesting a mechanism of action that requires stringent structural constraints. Several hypotheses about the functions of Ngb have been put forward over and above O_2_ binding and transport: (i) it may act as a cytochrome *c* reductase, (ii) it may be involved in a signal transduction pathway by controlling the dissociation of GDP from the G protein α subunit and (iii) it may scavenge damaging oxygen or nitrogen radicals, for example under normoxia it may detoxify a harmful excess of nitric oxide (NO) to nitrate (NO_3_^−^), while under hypoxia it may produce NO from nitrite (NO_2_^−^) for blood-pressure control (Jin *et al.*, 2008 ▸; Tiso *et al.*, 2011 ▸; Ascenzi *et al.*, 2014 ▸).

In spite of its low similarity in sequence (less than 25%) relative to Mb and Hb (Burmester *et al.*, 2000 ▸), Ngb displays the typical globin fold (a 3/3 α-helical structure) and retains the key structural determinants of globins: the proximal His(F8)96 that coordinates the heme iron and the distal His(E7)64 that controls ligand affinity and selectivity at the sixth iron-coordination position (Dewilde *et al.*, 2001 ▸; Guimarães *et al.*, 2014 ▸; Pesce *et al.*, 2004 ▸; Vallone, Nienhaus, Brunori *et al.*, 2004 ▸). Notably, Ngb is also a hexacoordinate protein in the ‘deoxy’ ferrous form, with the rate-limiting step for ligand binding being the spontaneous breakage of the distal His(E7)64–heme bond.

It has been shown by crystallography (Vallone, Nienhaus, Matthes *et al.*, 2004 ▸) that a conformational transition involving a sliding of the heme has to take place in order to create the space to accommodate an external ligand, which is a unique mechanism for ligand-affinity modulation in the globin family. This heme sliding counterbalances the presence of internal coordination, leading to a different protein conformation and promoting the accessibility of the heme pocket to O_2_ and other external ligands (Avella *et al.*, 2014 ▸; Exertier *et al.*, 2019 ▸).

**Figure 1:**
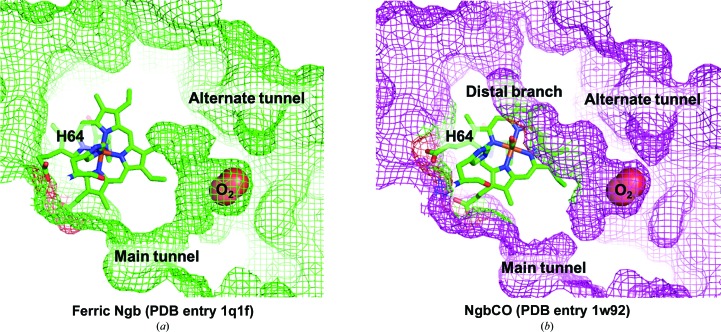
Large hydrophobic cavity and O_2_-docking sites in Ngb. The determination of neuroglobin structures in 2004 unravelled the existence of a large hydrophobic internal cavity in which the heme is accommodated, along with the main ‘tunnel’ and an alternate tunnel connecting this cavity to the bulk. The internal cavity and tunnels are shown in green (*a*) for hexacoordinate ferric Ngb (PDB entry 1q1f; Vallone, Nienhaus, Brunori *et al.*, 2004 ▸) and in magenta (*b*) for CO-bound Ngb (PDB entry 1w92; Vallone, Nienhaus, Matthes *et al.*, 2004 ▸), in which the location of O_2_ has been modelled within the cavity.

Ngb is endowed with a large hydrophobic cavity (300 Å^3^) and a substantial ‘tunnel’ displaying two branches, which extend around the heme and connect the distal and the proximal sides of the heme pocket, together with an alternate tunnel (Fig. 1 ▸; Colloc’h *et al.*, 2008 ▸; Vallone, Nienhaus, Brunori *et al.*, 2004 ▸). The heme sliding coupled to ligand binding affects the topology of the cavity, drastically reducing the proximal branch and enlarging the distal branch. In analogy to the internal packing defects detected in Mb, this tunnel implies an offset in terms of thermodynamic stability. Since cavities play a functional role in Mb by regulating the dynamics of ligand binding (Brunori & Gibson, 2001 ▸; Schlichting & Chu, 2000 ▸), it has been proposed that the Ngb tunnel, which represents an amplification of the set of Mb internal cavities, may play a similar role (Moschetti *et al.*, 2009 ▸; Colloc’h, Carpentier *et al.*, 2017 ▸). This hypothesis was supported by molecular-dynamics (MD) simulations, showing the similarity between the cavity system in Ngb and the xenon sites in Mb (Anselmi *et al.*, 2007 ▸), since during MD trajectories they both host ligands and constitute a preferential pathway for migration within the protein. High hydrostatic pressure also reinforces this hypothesis, showing that Ngb would hinge around a mechanical nucleus of five hydrophobic residues (Val68, Ile72, Val109, Leu113 and Tyr137) lining the cavity just behind the heme (Colloc’h, Sacquin-Mora *et al.*, 2017 ▸). Mutation studies have indeed highlighted the possibility that these residues could be involved in ligand migration (Astudillo *et al.*, 2012 ▸; Tejero *et al.*, 2015 ▸).

In globins, the network of cavities has been investigated extensively to characterize their role and dynamics, and it was shown that xenon docks into these apolar niches (de Sanctis *et al.*, 2004 ▸; Milani *et al.*, 2004 ▸; Moschetti *et al.*, 2009 ▸; Savino *et al.*, 2009 ▸; Tilton *et al.*, 1994 ▸; Abraini *et al.*, 2014 ▸). Moreover, direct crystallographic detection of CO in the protein matrix was reported in Hb and Mb by cryotrapping using helium cryostats under photodissociating conditions (Adachi *et al.*, 2003 ▸; Brunori, 2000 ▸; Schlichting *et al.*, 1994 ▸; Schmidt *et al.*, 2005 ▸). Time-resolved Laue crystallography at synchrotrons (Bourgeois *et al.*, 2006 ▸) and XFELs (Barends *et al.*, 2015 ▸) unveiled the subtle protein dynamics involved in ligand migration of photodissociated CO into Mb cavities on fast and ultrafast timescales, respectively. In solution, time-resolved spectroscopy detected the presence of ligand-docking intermediates in Mb (Lim *et al.*, 1997 ▸; Nienhaus *et al.*, 1994 ▸), and *in silico* MD simulations analysed the presence of transient cavities and gaseous ligand-migration pathways (Amadei & Vallone, 1996 ▸; Bossa *et al.*, 2005 ▸; Elber & Karplus, 1987 ▸). The general picture that emerged from several experimental and theoretical approaches on globins is that cavities modulate ligand kinetics and therefore protein reactivity.

In the case of Ngb, the presence of a heme iron-bound distal His(E7)64 and of the exogenous ligand-linked ‘heme sliding’ structural transition demand *ad hoc* investigation to evaluate the internal structural dynamics and the migration pathways of small gaseous ligands (CO and O_2_). In this work, the problem of the function of internal cavities is approached using several concurrent methodologies, *i.e.* UV–visible single-crystal microspectrophotometry to investigate the *in crystallo* optical spectra of NgbCO, X-ray diffraction (XRD) and X-ray absorption near-edge structure spectroscopy (XANES) at low temperatures to determine the three-dimensional structure of NgbCO and of the photoproduct Ngb*CO (obtained either by steady visible illumination in the crystal or by long X-ray exposure in solution). Moreover, the three-dimensional structure of ferric Ngb (not competent for CO/O_2_ binding) under increasing O_2_ pressure was investigated in order to find out whether internal cavities in Ngb may act as storage niches for this small gaseous reactant as observed when using Xe or Kr as probes (Colloc’h *et al.*, 2008 ▸; Duff *et al.*, 2004 ▸; Lafumat *et al.*, 2016 ▸).

## 2. Experimental procedures   

### 2.1. Protein purification and crystallization   

The expression, purification and crystallization of recombinant murine ferric Ngb (with Ser55 and Ser120 mutated to Cys) were carried out as described by Arcovito *et al.* (2007 ▸).

Crystals of the NgbCO derivative were obtained as reported previously (Vallone, Nienhaus, Matthes *et al.*, 2004 ▸) by treating ferric Ngb crystals with sodium dithionite to obtain the ferrous form competent for CO binding and then soaking them in CO-saturated mother liquor. Crystals that undergo this treatment have to be promptly cooled as they tend to dissolve upon prolonged exposure to CO.

### 2.2. *In crystallo* UV–visible microspectroscopy at 15 and 30 K   

Single NgbCO crystals were analysed by UV–visible microspectroscopy. The UV–visible microspectrophotometer includes a detector that can perform both absorption and fluorescence measurements and is available at the Cryobench of the European Synchrotron Radiation Facility (ESRF), Grenoble, France. A detailed description of the apparatus, the setup of which does not involve a polarizer, has been reported (Royant *et al.*, 2007 ▸; von Stetten *et al.*, 2015 ▸).

In conjunction with this configuration, an open-flow helium cryostat (Helijet, Oxford Diffraction) was used, allowing the collection of data in a temperature range between 15 and 30 K. This apparatus was modified to minimize ice formation during data collection and to achieve better temperature stability (McGeehan *et al.*, 2009 ▸; van der Linden *et al.*, 2013 ▸).

### 2.3. X-ray diffraction of NgbCO at 15 and 40 K with and without illumination   

X-ray data collections at low temperature were carried out on the ID14-2 beamline at ESRF at a wavelength of 0.933 Å using an ADSC Q4 CCD detector. The temperature was set in a range between 15 and 40 K using the Helijet connected to a temperature controller, instead of the standard nitrogen cryostream. Cooled NgbCO crystals were mounted onto cryo-loops and several diffraction data sets were collected from each sample at 40 K without illumination (‘dark’ state) and at 15 K under illumination (‘light’ state) by using an optical fibre microscope lamp, which was compatible with Helijet flow stability, whereas laser illumination geometry caused severe icing of the crystal and was thus avoided. With the aim of determining the structure of the photodissociation intermediate of NgbCO, we collected data from several crystals in different experimental sessions by illuminating them before (30 min) and during data collection. All diffraction data sets were processed using *MOSFLM* (Leslie, 2006 ▸) and the structures were refined with *REFMAC*5 (Murshudov *et al.*, 2011 ▸) from the *CCP*4 suite of programs (Winn *et al.*, 2011 ▸). Refinement statistics are reported in Table 1 ▸.

Additional ‘light–dark’ recordings confirmed that full rebinding was achieved by collecting ‘dark’ data after a photolyzed data set had been acquired (data not shown).

### 2.4. X-ray absorption near-edge spectroscopy (XANES) measurements of NgbCO in solution at 15 and 100 K   

The previously reported Fe *K*-edge XANES spectra of NgbCO (Arcovito *et al.*, 2008 ▸) were subjected to a different analysis that followed the *MXAN* procedure (Benfatto & Longa, 2001 ▸), which is usually applied to normalized absolute spectra, in order to extract structural information on the Ngb photoproduct.

The Ngb*CO–NgbCO XANES difference spectrum (Arcovito *et al.*, 2008 ▸) was fitted and a structure of the metal site of the Ngb*CO adduct was modelled using the following steps: (i) a reference XANES spectrum of NgbCO was calculated based on the NgbCO coordinates (PDB entry 1w92; Vallone, Nienhaus, Matthes *et al.*, 2004 ▸) and using the real part of the Hedin–Lundqvist potential without including any damping factor, (ii) at each step the *MXAN* package calculated the undamped Ngb*CO theoretical spectrum, using the Mb*CO coordinates (PDB entry 1abs; Schlichting *et al.*, 1994 ▸), while varying the selected coordinate parameters shown in Table 3 and (iii) finally, the damping factors were convoluted and directly fitted to the experimental Ngb*CO–NgbCO difference spectrum.

### 2.5. X-ray diffraction (XRD) of ferric Ngb under 50 and 80 bar O_2_ pressurization at 100 K   

Crystals under high dioxygen pressures (50 and 80 bar) were prepared using the soak-and-freeze technique as described previously (Lafumat *et al.*, 2016 ▸). Diffraction data were collected during two consecutive runs on the BM14 and BM30A (FIP) beamlines at ESRF under cryogenic conditions (100 K) at wavelengths of 0.8859 and 0.8559 Å for the 50 and 80 bar pressurized crystals, respectively. All data were processed with *XDS* (Kabsch, 2010 ▸). The *CCP*4 program suite was used for subsequent scaling, merging and structure refinements. All crystals were isomorphous to the ferric Ngb crystals reported previously (Vallone, Nienhaus, Brunori *et al.*, 2004 ▸). The models were subjected to iterative rounds of refinement and model building. Refinements were carried out with *REFMAC*5 (Murshudov *et al.*, 2011 ▸) from *CCP*4, followed by model adjustments and water additions with *Coot* (Emsley *et al.*, 2010 ▸). The quality of the final models was analyzed using *PROCHECK* (Laskowski *et al.*, 1993 ▸).

The atomic coordinates and structure factors have been deposited in the Protein Data Bank; the accession codes are reported in Table 1 ▸.

## 3. Results and discussion   

### 3.1. Characterization of NgbCO photolytic intermediates   

The complexity of CO rebinding in Ngb after photolysis has been described and attributed to CO migration from internal docking sites (Abbruzzetti *et al.*, 2009 ▸; Nienhaus & Nienhaus, 2004 ▸). Moreover, Nienhaus and coworkers investigated the ligand-binding reaction of NgbCO occurring after photolysis over a wide temperature range (3–353 K) using infrared and nanosecond time-resolved visible spectroscopy (Kriegl *et al.*, 2002 ▸). They reported that photolysis at cryogenic temperatures populates a pentacoordinate ‘deoxy’ ferrous species (Ngb*CO) with very low geminate rebinding barriers; this state can be trapped below 40 K, since at higher temperatures geminate rebinding becomes predominant. Therefore, we characterized single crystals of NgbCO by UV–visible optical microspectroscopy below 40 K, and in parallel we determined the structure of NgbCO at 15 K under continuous visible-light illumination using the ESRF Cryobench (Royant *et al.*, 2007 ▸; von Stetten *et al.*, 2015 ▸). The heme environment of NgbCO was characterized by XANES measurements in solution at 15 K under long X-ray exposure.

#### 3.1.1. UV–visible microspectrophotometry on NgbCO crystals at 15 and 30 K   

Ferric (Fe^3+^), ferrous (Fe^2+^) and ferrous CO-bound (Fe^2+^-CO) crystals of Ngb were mounted onto cryo-loops and flash-cooled in liquid N_2_ (100 K). The crystal orientation was optimized to maximize the signal from the heme metal centre while minimizing the baseline absorbance.

**Figure 2:**
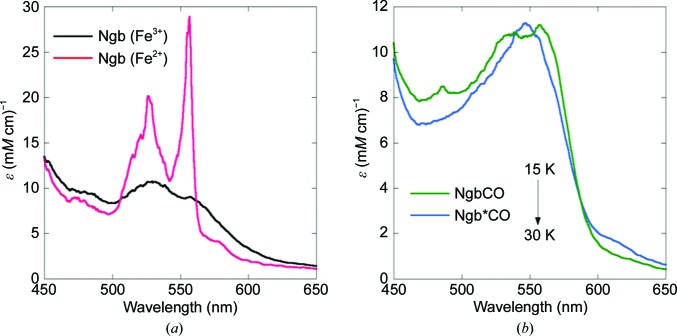
Absorption spectra of Ngb *in crystallo* at 15 and 30 K. (*a*) *In crystallo* UV–visible spectra of ferric (Fe^3+^) Ngb (black line) and hexacoordinate ferrous (Fe^2+^) Ngb (red line) were recorded at 15 K. Hexacoordinate ferrous (Fe^2+^) Ngb crystals were obtained by soaking in mother liquor containing sodium dithionite, a reducing agent. (*b*) It is possible to trap the photolytic intermediate Ngb*CO (cyan line) at 15 K under continuous illumination using the probing lamp of the microspectrophotometer. The 15–30 K temperature jump is associated with the geminate recombination of CO to the heme Fe atom (green line).

The absorption spectra of Ngb crystals in the ferric and ferrous (reduced with sodium dithionite) states at 15 K display the typical features of ferric Ngb and ferrous bis-histidyl hexacoordinate Ngb [Fig. 2 ▸(*a*)] as previously observed in solution (Brunori *et al.*, 2005 ▸; Dewilde *et al.*, 2001 ▸).

As far as absorption spectroscopy is concerned, the Ngb*CO photolytic intermediate should correspond to a ferrous (Fe^2+^) pentacoordinate species that differs from the ferric (Fe^3+^) and ferrous (Fe^2+^) hexacoordinate species, as shown in Fig. 2 ▸(*a*). Indeed, the spectrum obtained on exposing NgbCO crystals to continuous light at 15 K [Fig. 2 ▸(*b*), blue line] displays a single peak at 547 nm that corresponds to a ferrous pentacoordinate state, which has never so far been reported for wild-type Ngb; this shows that the continuous halogen lamp of the microspectrophotometer is not only a probe but is sufficient to induce photolysis, yielding the formation of a ferrous pentacoordinate Ngb*CO species at 15 K.

In order to demonstrate that the CO ligand is still trapped in the protein moiety of the Ngb*CO intermediate species, we increased the temperature to 30 K, where geminate rebinding becomes dominant. Accordingly, the resulting spectrum displayed two peaks (∼540 and ∼560 nm): the spectroscopic signature of Ngb bound to CO [Fig. 2 ▸(*b*), green line] (Dewilde *et al.*, 2001 ▸).

Finally, in order to confirm that the spectrum reported in Fig. 2 ▸(*b*) (blue line) unequivocally corresponds to that of Ngb*CO, we tested the reversibility of the whole process by proceeding with cycles of temperature increase from 15 to 30 K and decrease to 15 K (data not shown). The transition from the Ngb*CO photolytic intermediate at 15 K to CO-bound Ngb at 30 K was shown to be reversible in the course of several cycles. At room temperature the spectrum of ferrous Ngb corresponds to that of a hexacoordinate state with the distal histidine occupying the sixth coordination position. Thus, by trapping the Ngb*CO pentacoordinate state of reduced Ngb in the crystal at <30 K we demonstrated the feasibility of determining the three-dimensional structure of this photolytic intermediate.

#### 3.1.2. XRD structures of Ngb at 40 K and of the Ngb*CO photolytic intermediate at 15 K   

Crystal structures of NgbCO in the ‘dark’ state (no illumination) at 40 K and of Ngb*CO obtained under continuous illumination (‘light’ state) at 15 K were determined. Diffraction and refinement statistics are reported in Table 1 ▸.

**Figure 3:**
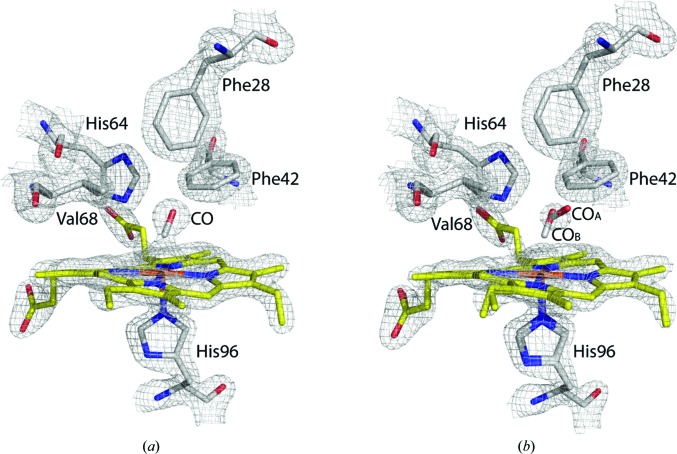
XRD electron-density maps of NgbCO at 40 K and of Ngb*CO at 15 K. (*a*) Close-up view of the heme pocket of NgbCO in the crystal structure determined at 40 K without illumination, showing a 2*F*_o_ − *F*_c_ electron-density map contoured at 1.5σ. The occupancy of CO bound to the heme was estimated to be 90%. (*b*) Close-up view of the heme pocket of Ngb*CO under illumination at 15 K, showing the electron-density map contoured at 1.5σ. The occupancy of CO_A_ is 40%, whereas that of CO_B_ was estimated to be about 10%.

The electron-density maps around the Ngb heme were contoured at 1.5σ and are shown in Fig. 3 ▸. Upon refinement of the ‘dark’ state NgbCO, the occupancy of heme-bound CO is estimated to be about 90%. The partial CO occupancy is owing to the intrinsic instability of CO-bound Ngb crystals, which tend to dissolve upon full ligation. We therefore avoided prolonged CO soaking, with the drawback of not achieving full ligation. In this work, we only utilized crystals with at least 90% CO ligation, as assessed by its occupancy in the determined structures [Fig. 3 ▸(*a*)].

Under continuous illumination, the electron density for the heme group indicates the existence of a minor Ngb population in which CO is still bound to the heme [10% occupancy; CO_B_ in Fig. 3 ▸(*b*)] and of a population in which CO is distinctly dissociated from the heme [40% occupancy; CO_A_ in Fig. 3 ▸(*b*)]. Therefore, a high photolysis yield was achieved with only 10% geminate rebinding (or partial photolysis) under continuous illumination, consistent with IR (Kriegl *et al.*, 2002 ▸) and visible spectroscopy at cryogenic temperature (reported above in Section 3.1.1). The minor fraction of CO-bound Ngb was either not detectable in the visible spectra or was caused by a temperature slightly higher than that achieved at the Cryobench, owing to the different sample geometry imposed by the diffraction data-collection setup.

The 40% occupancy of photodissociated CO is expected on the basis of Fourier transform infrared temperature-derivative spectroscopy data, indicating the presence of different subsites in the distal heme pocket (Lutz *et al.*, 2009 ▸). We therefore conclude that this 40% occupies a low-mobility subsite and that the remaining photodissociated CO molecules (50% of the total CO) reside in nearby subsites or migrate to docking sites further away within the protein matrix, in both cases being characterized by high mobility and therefore not detectable by XRD. The presence of photolysed CO in the protein matrix, which was not visible in the Ngb*CO structure, was inferred by the full recovery of bound CO in the ‘dark’ structures collected after illumination.

As shown in Fig. 3 ▸(*b*), the electron density is discontinuous between the iron and CO, indicating bond rupture *in crystallo* under continuous illumination at 15 K. In this Ngb*CO pentacoordinate intermediate, the photodissociated CO lies on top of the heme pyrrole C ring, in a position similar, but not identical, to the ‘primary docking site’ observed in sperm whale Mb (swMb; Schlichting *et al.*, 1994 ▸; Lim *et al.*, 1995 ▸). In the Ngb ‘primary docking site’, the distance between the iron and the carbonyl C atom (C_CO_) is about 2.7 Å, which is larger than the Fe—C_CO_ distance (1.9 Å) measured for the coordination bond in NgbCO structures at both 40 and 100 K (PDB entry 1w92; Vallone, Nienhaus, Matthes *et al.*, 2004 ▸). As a consequence of the photodissociation and of the subsequent CO displacement in the ‘primary docking site’, the distances between CO and residues lining the heme cavity changed, as reported in Table 2 ▸. Comparison with swMb*CO (Schlichting *et al.*, 1994 ▸) shows that in Mb the CO molecule moves towards the distal His(E7)63, whereas in Ngb*CO it is displaced in the opposite direction within the distal pocket owing to the bulk of His(E7)64. Notably, His(E7)64 stays put in Ngb crystals, while the heme itself slides deeper to make room for the ligand upon binding, whereas in swMbCO His(E7)63 swings towards the bulk.

The set of side chains to which the photodissociated CO is closest includes Phe(B10)28, Phe(CD1)42, His(E7)64, Val(E11)68 and Val(G8)109. This is consistent with the effect of an increase in the geminate rebinding fraction upon the mutation of Val68 to Phe, which would affect CO docking by steric hindrance (Astudillo *et al.*, 2012 ▸). Notably, all residues that form the surface for CO docking in Ngb, apart from His(E7)64, form a ‘mechanical nucleus’ that plays a key role in Ngb dynamics (Colloc’h, Carpentier *et al.*, 2017 ▸).

#### 3.1.3. Further analysis of the XANES spectra of NgbCO in solution under prolonged X-ray irradiation at 15 K   

X-ray-induced photolysis has been characterized for swMbCO, for which the X-ray-induced and light-induced photoproducts at low temperatures are alike, suggesting that CO photolysis by prolonged X-ray irradiation is a common feature of CO-bound heme proteins and yields comparable intermediate species (Della Longa & Arcovito, 2010 ▸; Milani *et al.*, 2008 ▸).

In a previous study, XANES spectra of NgbCO were collected at 15 K under X-ray exposure to investigate the Ngb*CO photolytic intermediate (Arcovito *et al.*, 2008 ▸). Starting from NgbCO in solution, a clear spectral evolution over time was observed when the beam spot was kept fixed, indicating that Fe—C_CO_ bond rupture was induced by X-rays. Moreover, the observed spectral evolution and the existence of at least three isosbestic points (at 7117, 7128 and 7142 eV) in the XANES profile indicated that the whole process accounted for a two-state transition involving the ferrous hexacoordinate NgbCO species and the ferrous pentacoordinate Ngb*CO species. Since the whole process has been shown to be reversible, this experimental setup allowed the acquisition of the XANES spectrum of a largely photolyzed Ngb*CO intermediate species in solution after prolonged X-ray exposure at 15 K. On the other hand, at 100 K, where CO geminate rebinding is much faster than the XANES acquisition time, only the fully CO-bound Ngb spectrum was recovered (NgbCO). Here, a further analysis of the abovementioned data allows us to determine the *ab initio* structure of the active site of the photolytic intermediate Ngb*CO species to complement the structure obtained by XRD.

**Figure 4:**
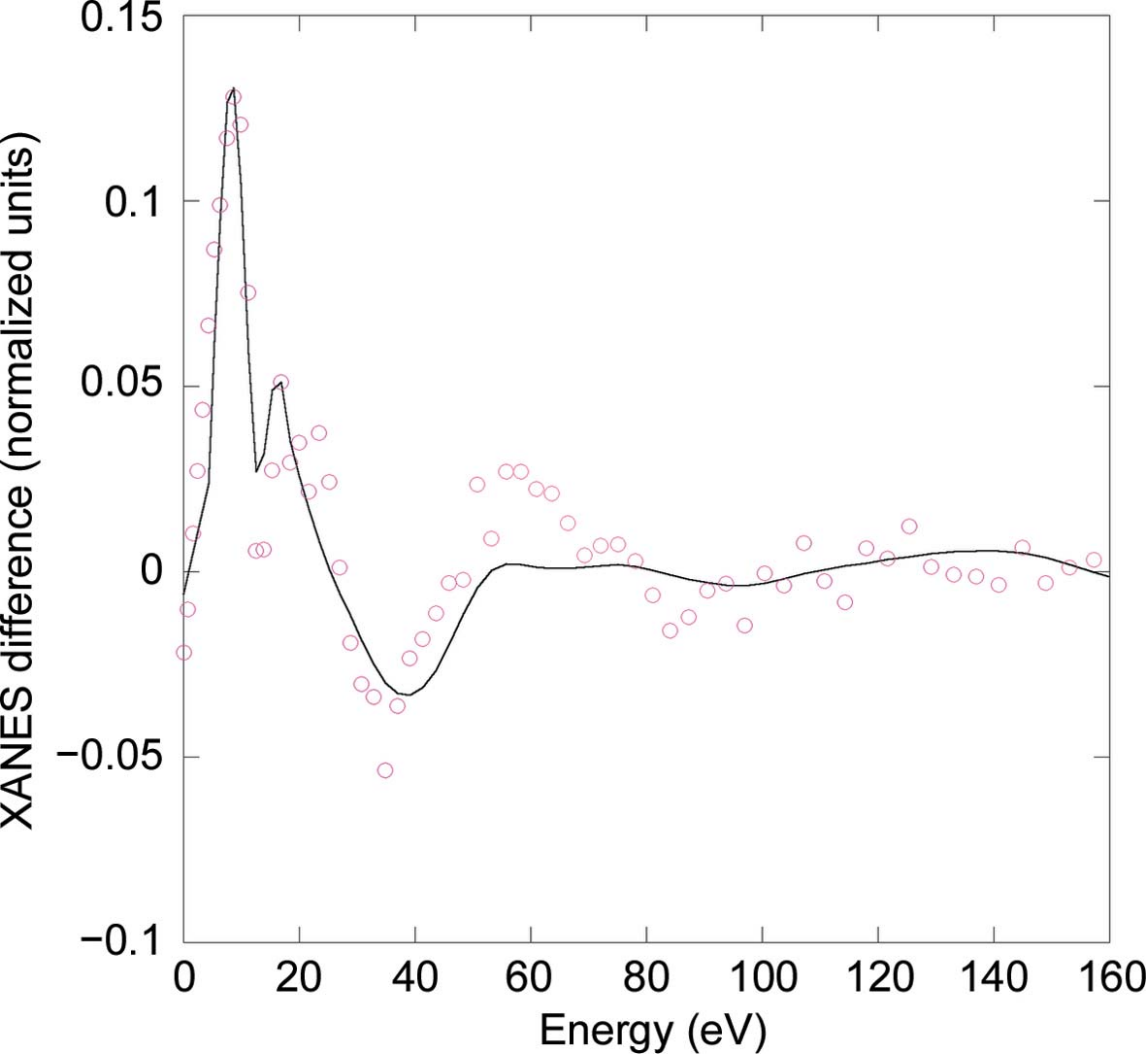
Further XANES analysis of the Ngb*CO–NgbCO difference spectrum at 15 K. The experimental Ngb*CO–NgbCO difference spectrum (red open circles; Arcovito *et al.*, 2008 ▸) was further analyzed and fitted (black solid line) using the *MXAN* software. Fitting parameters are reported in Table 3 ▸.

In previous work (Arcovito *et al.*, 2008 ▸), only the fit of the absolute species NgbCO was obtained using the *MXAN* procedure, while no attempt to fit the structure of the photoinduced Ngb*CO species in solution was performed, whereas in the new analysis presented here we applied the *MXAN* package tool to the Ngb*CO–NgbCO difference spectrum. According to this procedure, it is possible to determine the structure of the X-ray-induced photolytic intermediate, assuming that the structure of the NgbCO bound species is known and that only a single Ngb*CO species exists. Therefore, the *MXAN* package fits the experimental data in the form of a difference spectrum, the interpretation of which would provide a description of the iron environment of the photoproduct in solution (Benfatto & Longa, 2001 ▸; see Section 2). The resulting fit of the XANES difference profile is reported in Fig. 4 ▸ as a black continuous line, while the experimental difference spectrum is shown as red open circles. The fit is in overall agreement with the experimental data from 0 to 40 eV and deviates slightly at increased energies (Fig. 4 ▸), as indicated by root-mean-square deviations (r.m.s.d.s) ranging from 0.012 to 0.029 normalized units, in comparison to an estimated noise of 0.01 normalized units. Theoretical systematic errors in the XANES fitting have been thoroughly discussed (D’Angelo *et al.*, 2010 ▸; Arcovito *et al.*, 2005 ▸) and it appears that they can be reduced by the analysis of a difference spectrum, which we performed in the new analysis reported here. Systematic errors are mostly owing to the poor approximation used for the phenomenological broadening function Γ(*E*) adopted in the *MXAN* method (Benfatto & Longa, 2001 ▸), which mimics the electronic damping. However, systematic errors do not appreciably affect structural results, as has already been demonstrated for model systems such as Fe-porphyrin, Fe-heme and Fe(CN)_6_, confirming that XANES is more dependent on the geometry of the atomic cluster rather than its electronic structure (Arcovito *et al.*, 2005 ▸; D’Angelo *et al.*, 2008 ▸, 2010 ▸; Della Longa *et al.*, 2001 ▸, 2003 ▸, 2009 ▸; Hayakawa *et al.*, 2004 ▸; Lima *et al.*, 2014 ▸; Chillemi *et al.*, 2018 ▸). Notably, in our XANES analysis, the largest observed r.m.s.d. concerns the data obtained for energies ranging from 15 to 30 eV and we therefore cannot exclude other sources of systematic error. However, calculated systematic errors do not affect our overall conclusions since we could unambiguously distinguish between different degrees of iron displacement with respect to the heme or to the CO photoproduct in the Ngb*CO photolytic intermediate structure, as previously demonstrated for different light-induced sperm whale Mb*CO photoproducts, to which the same fitting procedure was applied (Arcovito *et al.*, 2005 ▸).

Fitting parameters from our new XANES analysis are reported in Table 3 ▸, together with those of the presently described XRD results and those derived from previously published XRD and XANES measurements on neuroglobin and myoglobin (Arcovito *et al.*, 2010 ▸). Analysis of the Ngb*CO–NgbCO difference spectrum shows (i) an iron doming suggested by a heme-iron displacement of 0.36 Å with respect to the 24-atom heme plane, similar to the displacement observed in Mb*CO, (ii) a distance of 2.04 Å between the heme iron and N_pyrrol_, which is in good agreement with that measured for Mb, (iii) an elongated distance between the heme and His(F8)96, yielding an Fe—N^∊^(His) distance of 2.24 Å, and (iv) a distance of 3.27 Å between the heme iron and the C atom of the photolyzed CO, which is similar to that reported for the Fe—*C_CO_ distance in Mb*CO (from both XRD and XANES measurements; Arcovito *et al.*, 2005 ▸; Schlichting *et al.*, 1994 ▸), but somewhat different from the Fe—*C_CO_ distance observed in Ngb*CO (2.7 Å) by XRD in this study. We ascribe this discrepancy to the 10% NgbCO bound species present in the crystals upon visible-light illumination that may affect the estimate of the Fe—*C_CO_ distance, owing to the lower signal provided by the CO photolysed species. Therefore, performing an *MXAN* analysis on an XANES difference spectrum allows a more accurate determination of the Fe—*C_CO_ distance to be obtained, since the fit isolates the contribution of the pentacoordinate Ngb*CO species.

Utilizing different illumination protocols or a customized light source compatible with the geometry of the helium cryostat could, in principle, improve the CO photolysis yield for XANES and bring it to the higher fraction observed in helium-temperature XRD. Photolysis owing to sole X-ray illumination, even at 15 K, allows only the partial recovery of photolytic intermediates, in agreement with the low barriers to rebinding and the high geminate recombination observed for Ngb (Kriegl *et al.*, 2002 ▸).

### 3.2. Characterization of a storage site for gaseous species in ferric Ngb: XRD structures of ferric Ngb under 50 and 80 bar O_2_ pressure at 100 K   

The localization of small gaseous ligands such as CO and O_2_ in the protein matrix is a nontrivial experimental task and is often inferred through indirect methods such as the use of xenon or halide anions as probes (Duff *et al.*, 2004 ▸; Roeser *et al.*, 2007 ▸). In Ngb, the identification of potential small gaseous ligand-binding sites has been investigated using Xe, Kr and Ar as probes (Abraini *et al.*, 2014 ▸; Moschetti *et al.*, 2009 ▸). These studies showed that the main noble-gas binding site with the highest occupancy is the Xe-III site located within the large heme cavity, while the occupancy in the Xe-IV site located between the Xe-III site and the heme was always lower (for example, at 40 bar Xe, 55% in Xe-IV compared with 85% in Xe-III; Colloc’h, Carpentier *et al.*, 2017 ▸).

In this work, we used X-ray crystallography under a high pressure of O_2_ to directly detect the presence of docking sites for small diatomic gases.

In the past, accurate control of the gas pressure during diffraction experiments was only achievable during room-temperature data collection, thus limiting the maximum pressure to about 50 bar owing to the limited resistance of quartz capillaries (Colloc’h *et al.*, 2008 ▸). A recently developed technique consisting of cryocooling the crystal directly in the liquid phase of the pressurized gas under isobaric conditions allowed this limitation to be overcome and pressures of up to 100 bar to be reached, as long as both the gas and liquid phases coexist at the selected pressure (Lafumat *et al.*, 2016 ▸). Therefore, diffraction data for ferric Ngb (Fe^3+^) were collected at 50 and 80 bar of pure molecular oxygen. This species is not competent for gaseous ligand binding, but is structurally identical within experimental error to the ferrous (Fe^2+^) hexacoordinate species that can bind O_2_ or CO (Arcovito *et al.*, 2008 ▸).

**Figure 5:**
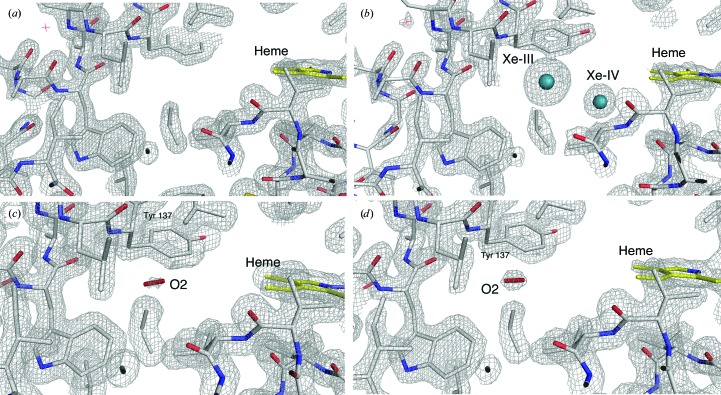
Oxygen-docking site in neuroglobin as determined by XRD at 50 and 80 bar O_2_. (*a*) The distal portion of the large internal cavity surrounding the heme as observed in the native ferric Ngb structure (PDB entry 1q1f; Vallone, Nienhaus, Brunori *et al.*, 2004). (*b*) Ferric Ngb at 30 bar Xe showing the two internal sites (called Xe-III and Xe-IV) in the distal portion of the large cavity from PDB entry 3gk9 or 4o4t (Abraini *et al.*, 2014 ▸; Moschetti *et al.*, 2009 ▸). (*c*, *d*) Ferric Ngb under 50 bar (*c*) and 80 bar (*d*) O_2_ showing the dioxygen molecule (50% occupancy at 50 bar versus 85% occupancy at 80 bar) only in the Xe-III site of Ngb. Electron-density maps are contoured at 1.5σ.

In spite of the limited equilibration time of the crystal in the gas phase (5 min) compared with the longer equilibration during RT experiments, one main O_2_-docking site is clearly observed at 50 and 80 bar O_2_ pressure, for which the occupancy increases from 50% at 50 bar to 85% at 80 bar [Figs. 5 ▸(*c*) and 5 ▸(*d*)]. This cavity corresponds to the Xe-III site (Abraini *et al.*, 2014 ▸; Colloc’h, Carpentier *et al.*, 2017 ▸; Colloc’h, Sacquin-Mora *et al.*, 2017 ▸; Moschetti *et al.*, 2009 ▸), which is devoid of electron density in all Ngb structures determined so far in the absence of pressurized gases [Figs. 5 ▸(*a*) and 5 ▸(*b*)]. Table 4 ▸ shows a list of the atoms lining the Xe-III niche and their distances from docked O_2_, within a radius of 5 Å. From this analysis, the hydrophobic nature of the Xe-III site surface is evident, since only two atoms out of 12 reported are polar. An analysis of the internal network of cavities in Ngb, carried out using coarse-grained Brownian dynamic simulations, high-pressure crystallography and mapping of noble-gas and nitrous oxide docking sites by XRD, led to the hypothesis that the Xe-III site acts as a storage site for small gaseous ligands (Abraini *et al.*, 2014 ▸; Colloc’h, Carpentier *et al.*, 2017 ▸; Colloc’h, Sacquin-Mora *et al.*, 2017 ▸; Moschetti *et al.*, 2009 ▸). This would be relevant for radical scavenging and, more generally, would support a catalytic role for Ngb. Indeed, as shown in Fig. 1 ▸, the Xe-III niche is located in the middle of the ‘tunnel’ connecting the bulk to the heme distal side and is sealed in the heme-slid NgbCO structure. We propose that the Xe-III site could host a diatomic molecule ready to react with an activated ligand bound to the heme, as observed for the reaction of NgbO_2_ with NO (Brunori *et al.*, 2005 ▸). Therefore, the presence of O_2_ in the Xe-III cavity supports its role as a ligand-storage site in the context of a catalytic role for Ngb. Our data show that none of the other cavities previously identified by Xe, Kr and Ar docking, including the Xe-IV site close to the heme cavity [Fig. 5 ▸(*b*)], is occupied by O_2_ even when the pressure is raised to 80 bar.

## 4. Conclusion   

Mapping docking sites inside a protein enables snapshots of events that govern its reactivity and dynamics to be obtained, and for neuroglobin it could provide relevant clues for pinpointing its mechanism of action and shedding light on its involvement in gas-sensing and radical scavenging. Therefore, we have used different biophysical approaches to investigate the structure of Ngb hosting diatomic gaseous molecules, such as CO and O_2_, which can dock in the large cavity surrounding the heme.

Notably, we combined ultralow-temperature X-ray diffraction and spectroscopy to determine the structure of the pentacoordinate Ngb*CO photolytic intermediate, with the latter method yielding a more accurate description of its geometry than XRD.

Molecular-dynamics simulations have predicted the trajectories of gas diffusion in and out of proteins (Shadrina *et al.*, 2016 ▸; Cohen *et al.*, 2008 ▸; Anselmi *et al.*, 2007 ▸). However, our experimental results, besides being relevant benchmarks for future simulations, directly provide a map of transient docking sites, which might represent structural snapshots of gas migration within neuroglobin.

By using complementary crystallographic approaches, *i.e.* ultralow-temperature and standard data collection at 100 K, we have singled out the location of gaseous ligands immediately after dissociation or approaching the heme iron site for binding. No secondary docking sites for photodissociated CO were detected in the distal heme side of neuroglobin at low temperature, or at least not with an affinity and a reduced mobility compatible with trapping and detection by cryo-crystallography. We do not exclude the potential design of mutations that would allow the detection of more elusive docking sites, as in the case of the myoglobin mutant Mb-YQR at 15 K (Brunori *et al.*, 2000 ▸) or Mb L29W at a temperature above 180 K (Ostermann *et al.*, 2000 ▸).

Moreover, the structure of ferric Ngb with internally docked O_2_ provides direct evidence for a ligand-storage location. These data, which show molecular oxygen hosted in the Xe-III site, confirm the predictive power of rare gases (Xe, Kr and Ar) for identifying docking sites and internal paths within proteins, but highlight the importance of validating them using physiologically relevant ligands. Indeed, only one (Xe-III) of the two internal Xe sites identified in Ngb hosts molecular oxygen and it coincides with the region of the Ngb cavity network that binds noble gases with the highest occupancy. We therefore hypothesize that it constitutes a preferential path for ligand migration and/or a storage site for diatomic molecules.

## Supplementary Material

PDB reference: Ngb under 50 bar O_2_ pressure, 5mjc

PDB reference: Ngb under 80 bar O_2_ pressure, 5mjd

PDB reference: NgbCO without illumination, 6i3t

PDB reference: NgbCO under illumination, 6i40

## Figures and Tables

**Table 1:** Crystallographic data-collection and refinement statistics for NgbCO and for ferric Ngb under 50 and 80 bar O_2_ Values in parentheses are for the outer shell.

	NgbCO (‘dark’) at 40 K	Ngb*CO (‘light’) at 15 K	Ferric under 50 bar O_2_ at 100 K	Ferric under 80 bar O_2_ at 100 K
PDB code	6i3t	6i40	5mjc	5mjd
Data collection				
Beamline	ID14-2, ESRF	ID14-2, ESRF	BM14, ESRF	BM30-A, ESRF
Wavelength (Å)	0.933	0.933	0.8959	0.8559
Resolution range (Å)	44.63–2.00 (2.10–2.00)	44.63–1.90 (2.00–1.90)	62.80–1.62 (1.67–1.62)	63.09–1.70 (1.75–1.70)
Space group	*R*32	*R*32	*R*32	*R*32
*a*, *b*, *c* (Å)	88.06, 88.06, 110.16	88.04, 88.04, 110.16	87.35, 87.35, 112.65	87.63, 87.63, 113.46
α, β, γ (°)	90, 90, 120	90, 90, 120	90, 90, 120	90, 90, 120
Unique reflections	11287 (1614)	12847 (1878)	19661 (1071)	16853 (830)
Multiplicity	7.1 (7.2)	3.7 (3.6)	7.3 (7.4)	7.7 (6.9)
Completeness (%)	99.9 (100.0)	98.5 (99.9)	99.6 (100.0)	94.7 (98.0)
Mean *I*/σ(*I*)	27.2 (7.5)	16.9 (3.0)	21.4 (4.3)	20.3 (3.8)
*R*_merge_ †	0.053 (0.236)	0.055 (0.373)	0.069 (0.281)	0.045 (0.290)
Refinement				
Resolution range (Å)	36.04–2.00	36.03–1.90	30.0–1.62	20.0–1.70
*R*_work_ ‡	0.17	0.17	0.168	0.179
*R*_free_ §	0.22	0.21	0.207	0.242
No. of atoms				
Protein	1664	1715	1172	1172
Heme	43	43	43	43
Sulfate	—	—	5	5
Dioxane	—	—	12	—
CO or O_2_	2	2	2	2
FMT	9	9	—	—
ACT	16	16	—	—
GOL	12	12	—	—
Water	99	101	144	128
Thermal *B* factors (Å^2^)				
Protein	24.0	25.9	27.0	35.2
Heme	22.5	23.65	16.5	20.1
Sulfate	—	—	21.8	26.5
Dioxane	—	—	30.0	—
FMT	50.2	52.7	—	—
ACT	43.8	47.6	—	—
GOL	33.6	36.3	—	—
Ligand (CO or O_2_)	19.4	15.2	34.9	38.1
Water	33.0	34.5	38.1	42.7
Overall	24.8	26.7	27.4	34.8
R.m.s.d. from ideality				
Bond lengths (Å)	0.014	0.013	0.03	0.016
Bond angles (°)	1.467	1.373	2.3	1.78

† *R*_merge_ is defined as 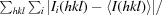 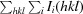, where *I*_i_(*hkl*) is the *i*th observation of reflection *hkl* and 〈*I_i_*(*hkl*)〉 is the weighted mean of all observations (after rejection of outliers).

‡ *R*_work_ is defined as 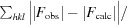 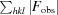 and indicates the accuracy of the model.

§ *R*_free_ is the cross-validation residual calculated using 5% of the data, which were randomly chosen and excluded from the refinement.

**Table 2:** XRD structures of NgbCO: distances between CO atoms and protein atoms In the second column we report the distances between CO and the atoms of its neighbouring residues measured for NgbCO (PDB entry 1w92; Vallone, Nienhaus, Matthes *et al.*, 2004 ▸) at 100 K and in the third column we report those observed for the ‘dark’ form of NgbCO at 40 K, while in the fourth column we report the distance between the photolyzed CO (CO_A_) and its neighbouring protein atoms for the ‘light’ form at 15 K. We only list distances of <5.0 Å for the atoms of the residues that are most involved in the ligand-migration process.

Neighbouring amino acid	NgbCO, 100 K, distance from CO atoms (C/O) (Å)	NgbCO, 40 K, distance from CO atoms (C/O) (Å)	Ngb*CO, 15 K, distance from *CO atoms (C/O) (Å)
Val68 C^γ2^	3.74/3.67	3.67/3.72	3.64/4.06
His64 C^∊1^	3.40/2.93	3.80/3.06	3.27/4.05
Phe28 C^ζ^	4.32/3.41	4.08/3.20	3.52/3.05
Phe42 C^ζ^	4.87/4.25	4.93/4.03	4.37/4.77

**Table 3:** Comparison of structural parameters determined by XANES and XRD for NgbCO and MbCO Estimated standard uncertainties for XRD were calculated using *DPIRfree* (Cruickshank, 1999 ▸). Values in parentheses represent the statistical error of the last digits.

Experiment	Fe–24-atom heme-plane displacement (Å)	Fe—N_pyrrol_ (Å)	Fe—His (Å)	Fe—C_CO_ (Å)
XRD, 2.0 Å, NgbCO at 100 K†	—	2.05 (20)	1.93 (22)	1.90 (21)
XANES (solution), NgbCO at 100 K‡	0.04 (fixed)	2.02 (2)	1.96 (7)	1.86 (5)
XRD, 1.9 Å, Ngb*CO at 15 K§	—	2.05 (18)	2.11 (20)	2.66 (15)
XANES (solution), Ngb*CO at 15 K§	0.36 (30)	2.04 (3)	2.24 (20)	3.27 (27)
XRD, 1.5 Å, Mb*CO at 20 K¶	0.2	1.97	2.25	3.60
XANES (solution), Mb*CO at 15 K††	0.40 (5)	2.04 (2)	2.07 (3)	3.23 (10)

† Vallone, Nienhaus, Matthes *et al.* (2004 ▸).

‡ Arcovito *et al.* (2010 ▸).

§ Present work.

¶ Schlichting *et al.* (1994 ▸).

†† Arcovito *et al.* (2005 ▸).

**Table 4:** Distances between the O1 and O2 atoms located in the storage site (Xe-­III) and protein atoms

Pressure	50 bar O_2_	80 bar O_2_
Neighbouring residue	Distances (O1/O2) (Å)	Distances (O1/O2) (Å)
Ile72 C^γ1^	4.51/4.27	4.86/4.21
1le72 C^γ2^	4.17/3.92	4.40/3.94
Ale75 C^β^	—/4.04	4.84/3.85
Leu113 C^δ2^	3.50/4.57	3.84/4.53
Trp133 C^ζ3^	3.61/4.10	3.66/4.21
Tyr137 C^α^	3.51/3.95	3.46/4.10
Tyr137 C^δ1^	3.65/4.28	3.98/4.33
Tyr137 N	3.65/4.04	3.54/4.29
Leu136 O	4.61/4.37	4.40/4.62
Leu136 C^δ2^	4.18/3.83	3.97/4.14
Leu136 C^γ^	4.02/3.66	3.59/3.81
Val140 C^γ2^	4.58/3.70	4.51/3.83

Only distances of <5.0 Å for O_2_ at 50 and 80 bar are listed.
